# A metagenomics-based approach to the top-down effect on the detritivore food web: a salamanders influence on fungal communities within a deciduous forest

**DOI:** 10.1002/ece3.1259

**Published:** 2014-10-08

**Authors:** Donald M Walker, Brandy R Lawrence, Dakota Esterline, Sean P Graham, Michael A Edelbrock, Jessica A Wooten

**Affiliations:** 1 Department of Natural Sciences, The University of FindlayFindlay, Ohio, 45840; 2 College of Pharmacy, The University of FindlayFindlay, Ohio, 45840; 3 The Department of Agricultural and Natural Resource Sciences, Sul Ross State UniversityAlpine, Texas, 79832; 4 Department of Biology, Centre CollegeDanville, Kentucky, 40422

**Keywords:** 454 pyrosequencing, decomposition, keystone species, *Plethodon cinereus*, Red-backed Salamander, soil community

## Abstract

The flow of energy within an ecosystem can be considered either top-down, where predators influence consumers, or bottom-up, where producers influence consumers. *Plethodon cinereus* (Red-backed Salamander) is a terrestrial keystone predator who feeds on invertebrates within the ecosystem. We investigated the impact of the removal of *P. cinereus* on the detritivore food web in an upland deciduous forest in northwest Ohio, U.S.A. A total of eight aluminum enclosures, each containing a single *P. cinereus* under a small log, were constructed in the deciduous forest. On Day 1 of the experiment, four salamanders were evicted from four of the eight enclosures. Organic matter and soil were collected from the center of each enclosure at Day 1 and Day 21. From each sample, DNA was extracted, fungal-specific amplification performed, and 454 pyrosequencing was used to sequence the nuclear ribosomal internal transcribed spacer (ITS2) region and partial ribosomal large subunit (LSU). Changes in overall fungal community composition or species diversity were not statistically significant between treatments. Statistically significant shifts in the most abundant taxonomic groups of fungi were documented in presence but not absence enclosures. We concluded that *P. cinereus* does not affect the overall composition or diversity of fungal communities, but does have an impact on specific groups of fungi. This study used a metagenomics-based approach to investigate a missing link among a keystone predator, *P. cinereus,* invertebrates, and fungal communities, all of which are critical in the detritivore food web.

## Introduction

The classic ecological study by Paine (1974) demonstrated that removal of a top predator (i.e., the starfish, *Pisaster ochraceus*) from tidal pools results in changes in community composition of lower trophic levels. Since this landmark study, the structure within diverse food webs has been shown to be influenced by both top-down (predators influencing consumers) and bottom-up effects (producers influencing consumers). The flow of energy from biomass in detrital food webs can affect both lower (bottom-up) and higher (top-down) trophic levels (Pace et al. 1999; Shurin et al. 2002). Saprophytic bacteria and fungi play a dominant role in determining energy flow among and within trophic levels (Crowther et al. 2013), especially in deciduous forests where nutrient and biomass inputs from the canopy support an inverted food web based upon decomposing leaf litter (Moore et al. 2003; Wardle 2006). Saprophytic microbes, such as cord-forming basidiomycetes, produce food stores for mycophagous (fungus-consuming) invertebrate communities, while both micro- and macro-invertebrates mix soil and shred leaf litter, allowing nutrients to be cycled through the detritivore food web (Crowther et al. 2013). In the eastern United States, the apex of this food web is occupied by a diverse and abundant assemblage of plethodontid salamanders. This study explores the top-down effect of a salamander (*Plethodon cinereus*) on the fungal community composition in a midwestern U.S. deciduous forest using a metagenomics-based approach.

**Figure 1:**
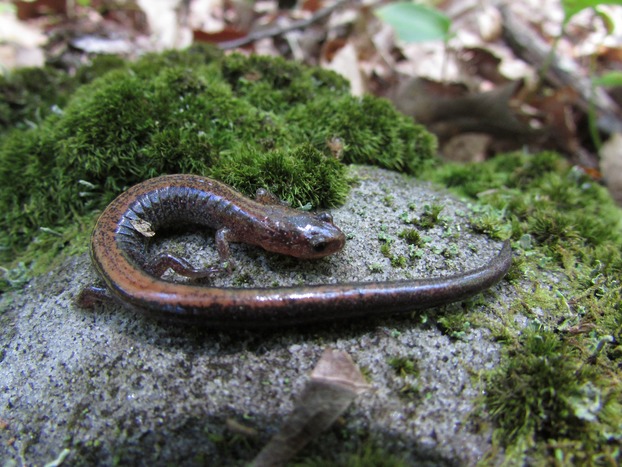
Digital image of the organism of study, *Plethodon cinereus*.

Due to their small size, local abundance, and amenability to field and laboratory research, the terrestrial plethodontid salamander species, *P. cinereus,* (Red-backed Salamander) has served as a model organism for studies of salamander behavioral ecology (Thurow 1975; McGavin 1978; Jaeger and Gergits 1979; Jaeger 1981) (Fig. 1). This species has been hypothesized to play an important role in the detritivore food web by exerting a significant top-down effect on lower trophic levels (Burton and Likens 1975; Hairston 1987; Petranka 1998; Davic and Welsh 2004). *Plethodon cinereus* are documented top predators of the detritivore food web in upland deciduous forests and exhibit large population sizes and biomass (Burton and Likens 1975). In fact, this single species was found to outweigh the passerine birds of a deciduous forest in New Hampshire (Burton and Likens 1975). *Plethodon cinereus* preys upon invertebrate communities, such as *Collembola* (springtails), *Diplopoda*, *Diptera*, isopods, mites, and spiders (Wyman 1998) that are integral members of decomposer ecosystems (Walton et al. 2006). Both macro- and micro-invertebrate communities are mycophagous on resident fungal communities involved in the detritivore food web. The Red-backed Salamander (juveniles, females, and males) exhibits territorial behavior and aggressively guards a home range between 12.87-24.34 m^2^ (Kleeberger and Werner 1982). They signal their presence using pheromones contained in fecal pellets, which deter other *P. cinereus* from intruding upon their foraging ground (Jaeger et al. 1986). Therefore, these small territories can be used as excellent replicated microhabitats to determine the effect of individual salamanders on the leaf litter microbial community.

The impact of keystone predators on lower trophic levels of the detritivore food web should be attenuated by a range of complex interactions including but not limited to species diversity, the heterogeneity of detritus supply/quality, prey species refugia, mutualism, or nonspecific prey selection by predators (Scheu and Setala 2002; Wardle 2002; Walton et al. 2006). Past studies have investigated the role of both *P. cinereus* and invertebrates (e.g., *Collembola*, isopods) in the detritivore food web including their effects on nutrient cycling and influence on decomposition rates of saprophytic microbes. These studies have documented various roles for *P. cinereus* including a reduction, increase, or no effect on invertebrate communities or the rate of decomposition in the detritivore food web (Wyman 1998; Rooney et al. 2000; Walton 2005; Walton and Steckler 2005; Walton et al. 2006; Homyack et al. 2010). These studies quantify the reported effects based upon insect morphology and invertebrate collections using various trapping methodologies. In this study, we used a molecular approach to compare fungal community diversity and assemblages in the absence/presence of *P. cinereus* within a natural environment. To our knowledge, this is the first study of its kind to use a metagenomic-based approach to explore the top-down effect of *P. cinereus* as a keystone species on fungal communities in the detritivore food web in nature.

The effects of *P. cinereus* were tested using 454 pyrosequencing to determine whether changes occur in fungal community composition or diversity after removing *P. cinereus* from enclosures in an upland deciduous forest of Ohio. We hypothesized that upon salamander eviction, isopods (and other invertebrates) are no longer under the predatory pressures of *P. cinereus* and are able to selectively ingest basidiomycete cords as in Crowther et al. (2013), thus reducing their abundance and opening new niches for ascomycete and zygomycete species. The objectives of this study were to (1) evaluate the dynamics of the detritivore food web with respect to the absence/presence of *P. cinereus* in an upland deciduous forest; and (2) determine whether *P. cinereus* exerts a top-down effect on fungal communities and the detritivore food web.

## Materials and Methods

### Study site and sampling

Samples were collected with permission from authorities at Litzenberg Memorial Woods in Findlay, Ohio (N: 41.06014; W: 083.76473). This study site is characterized by several small tributaries flowing through it from the Blanchard River and a mixed mesophytic deciduous forest dominated by *Acer saccharum, Fraxinus americana, Quercus alba,* and *Quercus rubra*. Eight 0.5 m^2^ aluminum flashing enclosures were erected on 26 August 2013; each enclosure contained a single *P. cinereus* individual found under a piece of decaying wood. The enclosures were 35 cm in height and buried at a depth of 15 cm to ensure that the salamander and invertebrate communities remained in the enclosure. The integrity of the natural environment was maintained during construction of the enclosures, and a 21-day “stabilization” period allotted before the first sample was taken. Organic matter containing decaying leaves, wood, and top 2–3 cm of soil (leaf litter and organic horizon) was haphazardly collected from the center of each of the eight enclosures at two different times (Day 1 and Day 21) over a 21-day period (Sample weight range = 19.5–41.6 g; mean weight = 28.1 g; *n* = 16). Samples were collected on Day 1 when each enclosure contained one salamander. Four salamanders were then evicted on Day 1 from each of four enclosures. A second organic matter sample was collected 21 days later from the four salamander absence and four presence enclosures. All 16 samples were stored at −80°C within 1 h of collecting until they were processed. This study used both presence–absence and before–after differences to explore the importance of salamander presence. The University of Findlay's institutional research policies and guidelines for the ethical treatment of animals were followed during this study.

### DNA extraction

Each sample containing the leaf litter, wood, and the organic horizon of the soil was homogenized for 60 sec using a Black & Decker HC306 1-1/2-Cup One-Touch Electric Chopper. The resulting particle size was approximately 1–3 mm. All components of the electric chopper were sterilized for 20 min in a 1:10 dilution of Clorox bleach and rinsed in molecular grade water between each sample. Total genomic DNA was isolated from 0.25 g wet weight of each sample (*n* = 16) using the MOBIO Power Soil DNA Extraction Kit (MOBIO Laboratories, Carlsbad, CA) according to the manufacturer's instructions and stored at −20°C.

### 454 pyrosequencing methods

The internal transcribed spacer (ITS) region of rDNA and partial ribosomal large subunit was amplified using the PCR primers ITS1F_KYO2 (Toju et al. 2012) and LR3 (http://biology.duke.edu/fungi/mycolab/primers.htm). Each PCR consisted of 12.5 *μ*L of HotStarTaq Master Mix (QIAGEN Inc, Valencia, CA), 1 *μ*L (10 *μ*mol/L) each primer, 9.5 *μ*L sterile DIH_2_O, and 1 *μ*L DNA. Thermal cycler conditions were as follows: 95°C for 15 min, followed by 20 cycles at 94°C for 20 sec, 50°C for 30 sec, 72°C for 120 sec, and final extension at 72°C for 7 min. Each PCR product was subjected to a second round of PCR that targeted the ITS2 region using 454 fusion primers in the following configuration: 5′ – 454 Adaptor A + 8-bp multiplex tag + forward gene-specific primer – 3′ and 5′ – 454 Adaptor B + reverse gene-specific primer. Each DNA extraction/PCR reaction representative of a single organic matter sample was tagged with a unique multiplex tag (MID; *n* = 16). The forward gene-specific primer was ITS3_KYO2 (Toju et al. 2012) and reverse primer LR_KYO1b (Toju et al. 2013). Each PCR consisted of 12.5 *μ*L of HotStarTaq Master Mix (Qiagen), 1 *μ*L (1 *μ*mol/L) each primer, 9.5 *μ*L sterile DIH_2_O, and 1 *μ*L PCR product. The thermal cycler conditions were as follows: 95°C for 15 min, followed by 40 cycles at 94°C for 20 sec, 50°C for 30 sec, 72°C for 60 sec, and final extension at 72°C for 7 min. The PCR products were purified twice with AMPureXP beads, analyzed for short fragments with qPCR, and quantified with the Invitrogen Qubit Fluorometer (Invitrogen, Carlsbad, CA) at Oregon State University (OSU) Center for Genome Research and Biocomputing. 454 pyrosequencing was performed on a GS Junior sequencer (Roche, Basel, Switzerland) at OSU. Negative controls were run during both DNA extraction and PCR.

### Sequence processing and analyses

**Figure 2:**
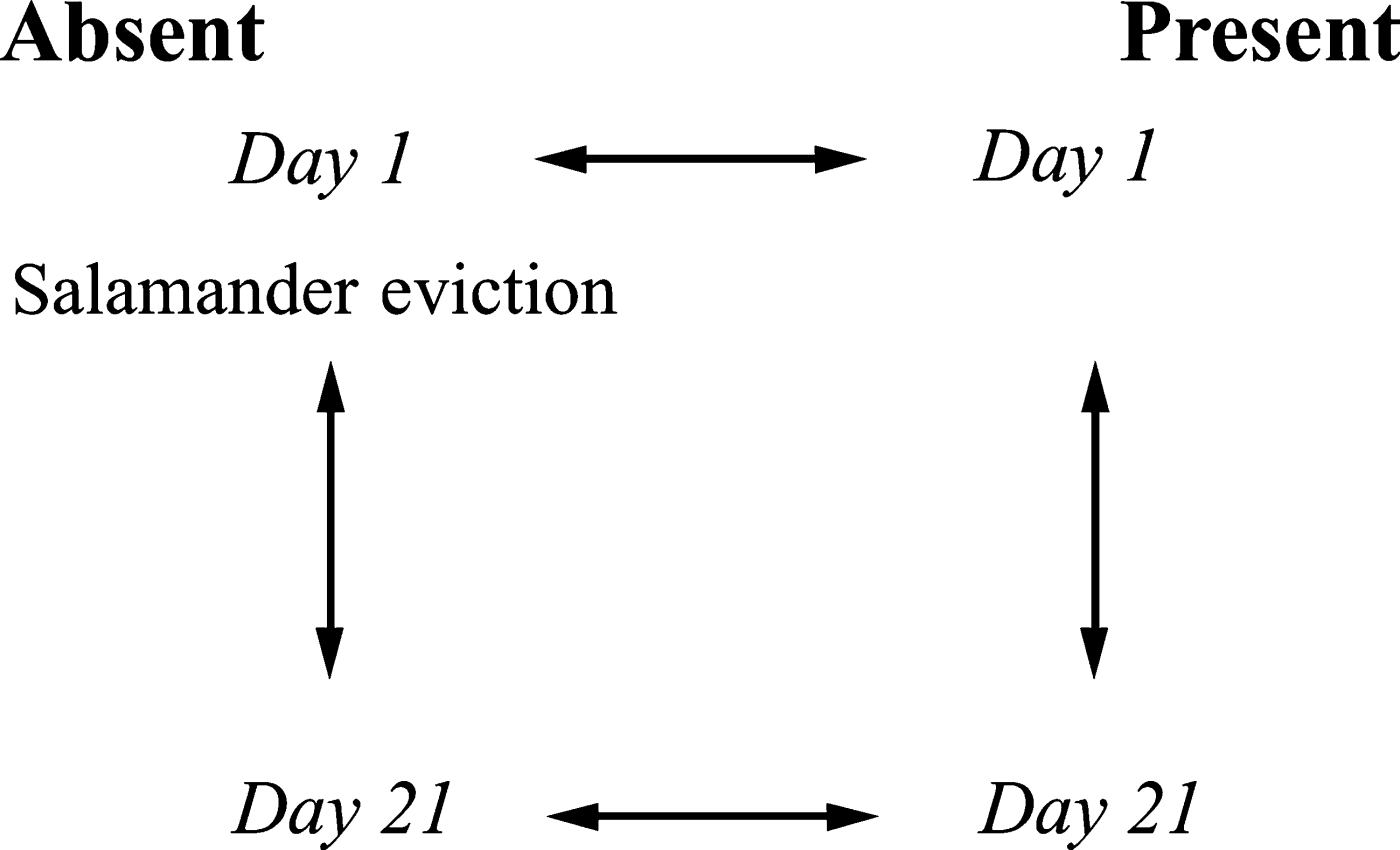
Soil sampling and analysis design. Each “*Day*” (Absent or Present) represents four soil samples taken from four independent enclosures. Each sample is encoded with a unique multiplex tag (MID) for analysis purposes. Arrows show independent analyses conducted in CloVR-ITS (e.g., comparison of four enclosures at Day 21 Absent versus four enclosures at Day 21 Present).

Sequencing resulted in 141,376 ITS sequences (GenBank Short Reads Archive: SRP045746). Reads were quality controlled using the PyroTrimmer software (Oh et al. 2012) under the following conditions: a minimum length of 150 bp, maximum length of 700 bp, average quality value cutoff of 20 for 3′ end trimming, average quality value cutoff of 25 for full-length sequences, 3 maximum mismatches for trimming primer sequences, 0 maximum ambiguous nucleotides, maximum homopolymer length of 8. Forward primers, MIDs, and reverse primers (if present) were removed before further analyses. All additional analyses were completed in CloVR-ITS (White et al. 2013), which is an automated pipeline for comparisons of ITS pyrosequencing data. Chimera detection and removal were completed in UCHIME (Edgar et al. 2011) according to White et al. (2013). All nonchimeric sequences were searched against the clovr-itsdb v1.0 curated database of ITS sequences (White et al. 2013) using BLASTN (Altschul et al. 1997) with an *e*-value cutoff of 1e-5. Each ITS sequence is assigned to a taxonomic group based on a minimum 90% query sequence length coverage and minimum identity of 75%, 70%, and 60% for family, order, and class rank, respectively (see White et al. 2013 for justification). Five total analyses were run using the CloVR-ITS automated pipeline. The first analysis included the complete curated (PyroTrimmer) data set consisting of 16 MIDs, which corresponds to a sample from a single enclosure (*n* = 8) at each sampling time (Day 1, 21; *n =* 16). The remaining four analyses are outlined in Figure 2 and compare four versus four enclosures at each of the two different sampling points.

### Diversity and statistical analyses

Taxonomic classification from BLASTN results in CloVR-ITS was processed using Metastats (White et al. 2009) under the default parameters to detect differentially abundant taxa at the class, order, and family ranks. *α* diversity analyses including rarefaction curves and the Shannon index were calculated in MOTHUR (Schloss et al. 2009). A two-way ANOVA was conducted using the Shannon index with sampling day (i.e., Day 1 or Day 21) and salamander presence or absence as the two variables using GraphPad Prism version 6.00 for Windows (GraphPad Software, La Jolla, CA; http://www.graphpad.com). The Shannon index has been shown to be normally distributed and homogeneous, meeting the assumptions of the two-way ANOVA. Sidak's multiple comparisons were used to determine any significant differences between day (i.e., Day 1 vs. Day 21) and presence versus absence of the salamander.

To determine differences in fungal community composition, the taxonomic assignment tables (e.g., class, order, family) from the CloVR-ITS analysis were standardized, square root-transformed, and a Bray–Curtis similarity index performed in PRIMER 6. The fungal community composition patterns of each enclosure at Days 1 and 21 were visualized using a nonmetric multidimensional scaling (MDS) ordination approach in PRIMER 6 with 50 restarts and a minimum stress of 0.01.

## Results

### 454 pyrosequencing results

Among the 141,376 raw ITS sequences, 40,574 reads were removed due to length, ambiguous bases, low-quality scores, and long homopolymers. An additional 6,979 reads were detected as chimeras in UCHIME (Edgar et al. 2011) and removed from the final data set. Eight soil samples (29,670 reads) were removed from the final data set that included 64,153 ITS sequences (range 150–576 bp; mean length 403 bp).

### Diversity results

Results of the two-way ANOVA suggest that there is no strong interaction between sample day and presence or absence of the salamander (*F*_1,12_ = 1.393; *P* = 0.261). There was no significant effect of day (*F*_1,12_ = 1.346; *P* = 0.269) or presence or absence of salamanders (*F*_1,12_ = 0.237; *P* = 0.6355). No other comparisons were significant between days or between presence and absence enclosures using Sidak's multiple comparisons post-hoc test indicating no significant difference in Shannon diversity indices.

### Fungal community structure

Reads from absence (*n* = 8) and presence (*n* = 8) enclosures make up 46.9% (31,834 reads) and 50.4% (32,319 reads) of the data set, respectively. (OTUs) Operational Taxonomic Units that fall into the “unclassified” category make up 34.02% (21,827 reads) of the entire data set. Overall, the phylum Ascomycota represents a larger fraction of the data set (45.5%; 29,199 reads) than the Basidiomycota (20.1%; 12,911). The five most abundant taxonomic classes throughout sampling space and time are the Agaricomycetes (19.7%; 12,604 reads), Leotiomycetes (14.5%; 9,315 reads), Dothideomycetes (15.3%; 9,816 reads), Sordariomycetes (9.9%; 6,373), and Eurotiomycetes (4.4%; 2,189 reads; Table 1). Species in the Agaricomycetes increased in absence enclosures from Days 1 to 21 (25–38.6% of reads), but decreased slightly in presence enclosures (14.7–12.4% of reads). There was an increase in the Dothidiomycetes in presence enclosures from Days 1 to 21 (16.5–28.5% of reads), while species in this class decreased slightly in absence enclosures (9.2–8.1%). The Sordariomycetes decreased from Days 1 to 21 in both presence (12–4.1% of reads) and absence enclosures (19.8–10.9% of reads).

**Table 1:** A summary of the top five most abundant fungal classes in absence and presence enclosures. Determined by pooling reads for each of four enclosures together at two different sampling times and dividing by the total number of reads for independently pooled samples

Class	Day 1				Day 21			
	
	Absence enclosures		Presence enclosures		Absence enclosures		Presence enclosures	
			
	# Reads	% Total reads	# Reads	% Total reads	# Reads	% Total reads	# Reads	% Total reads
Agaricomycetes	3355	25.0	2471	14.7	5179	38.6	2075	12.4
Dothideomycetes	1232	9.2	2776	16.5	1084	8.1	4778	28.5
Eurotiomycetes	451	3.4	886	5.3	422	3.1	416	2.5
Leotiomycetes	2586	19.3	1240	7.4	2555	19.1	3186	19.0
Sordariomycetes	2652	19.8	2014	12.0	1459	10.9	684	4.1

The rarefied data for Day 1 indicated 189 OTUs on average in absence and 190 OTUs in presence enclosures. On Day 21, the average number of OTUs in absence enclosures decreased (*n* = 172), while those in presence enclosures remained nearly the same (*n* = 189).

### *Plethodon cinereus* influence on fungal community structure

**Figure 3:**
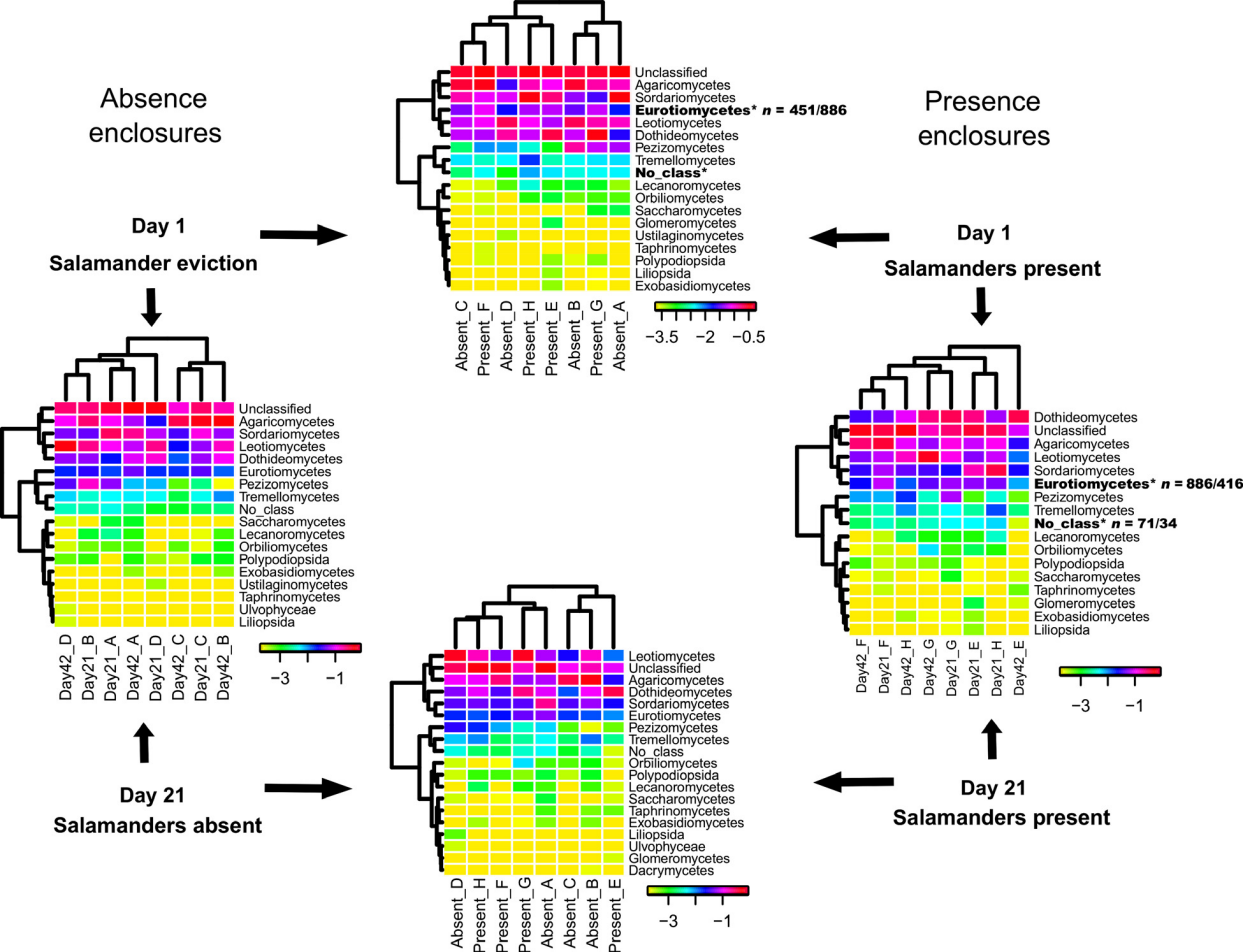
Log-transformed heat map at the taxonomic rank of class. *Bold text indicates a statistically significant change in fungal class (*P* < 0.05). *n* = #/# indicates the number of sequence reads associated with Day1/Day21 or Day1 Absent/Day1 Present, etc. The letters (ex. Absent_A or Day1_A) indicate a single enclosure sampled two times over the length of the experiment.

**Figure 4:**
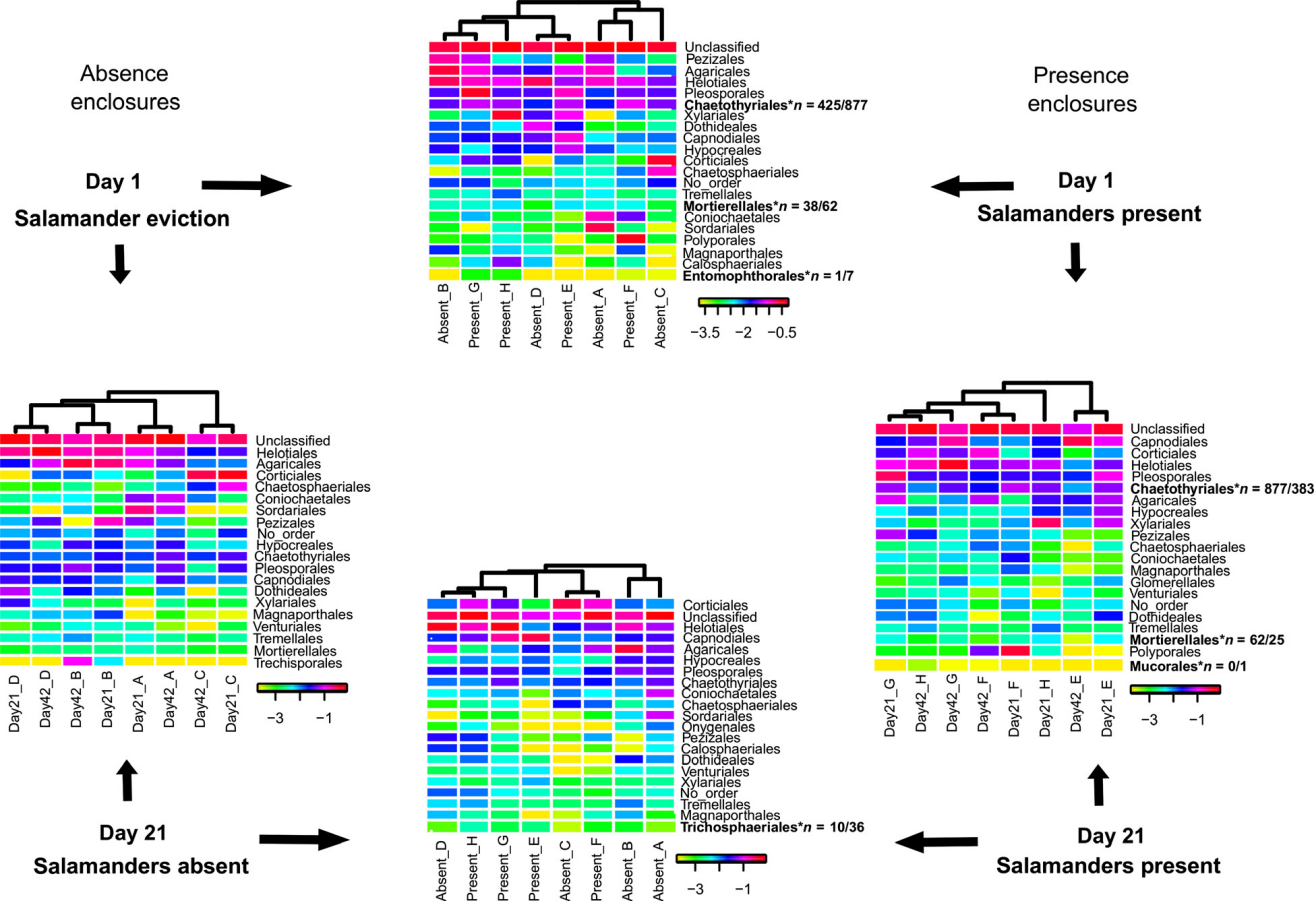
Log-transformed heat map at the taxonomic rank of order. Comparisons are based on total fungal diversity. Only the 20 most abundant phylotypes and any additional orders identified as statistically significant in Metastats are listed. *Bold text indicates a statistically significant change in fungal order (*P* < 0.05) through time or space. *n* = #/# indicates the number of sequences associated with Day1/Day21 or Day1 Absent/Day1 Present, etc. The letters (e.g., Absent_A or Day1_A) indicate a single enclosure sampled two times over the length of the experiment.

**Figure 5:**
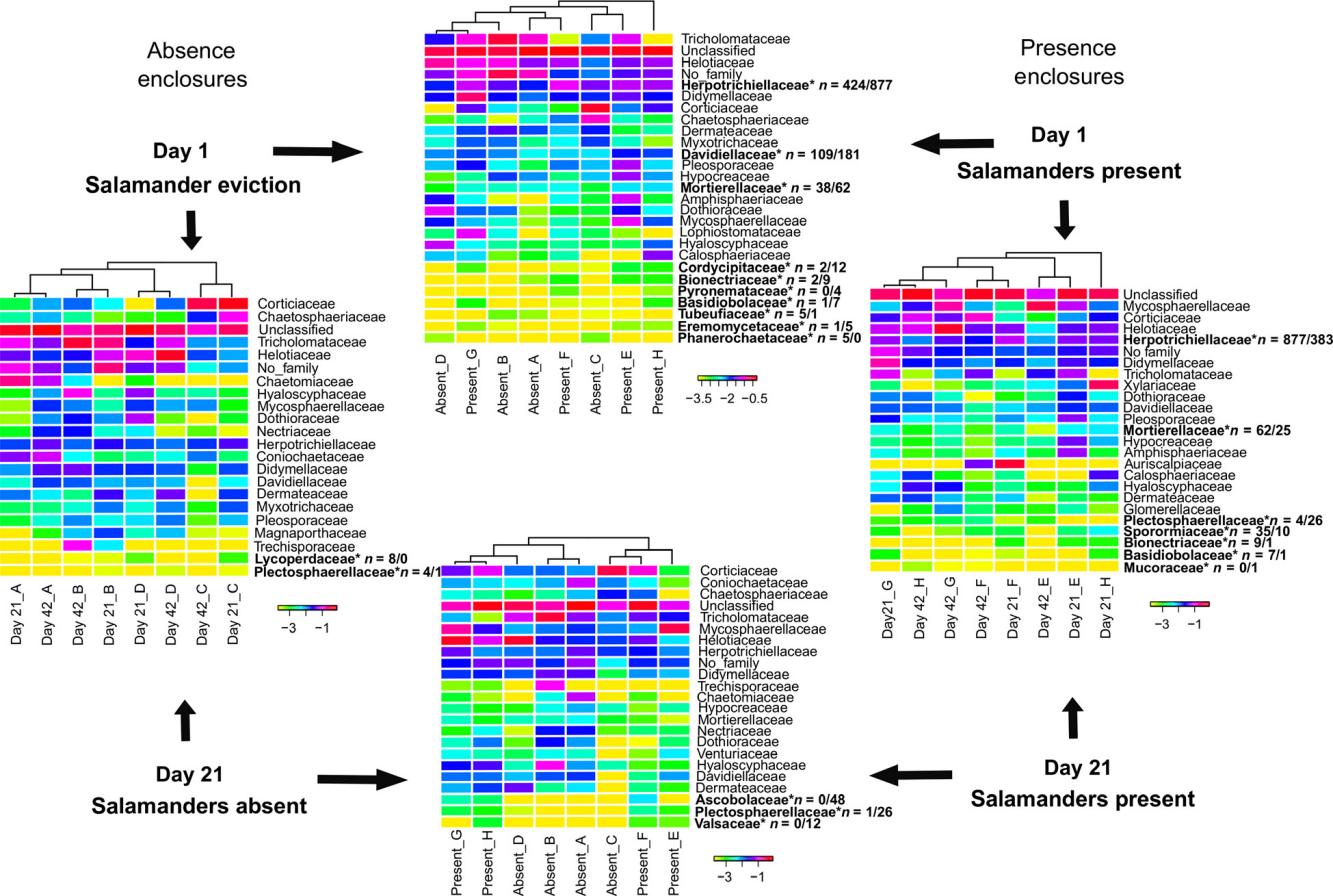
Log-transformed heat map at the taxonomic rank of family. Comparisons are based on total fungal diversity. Only the 20 most abundant phylotypes and additional families identified as statistically significant in Metastats are listed. *Bold text indicates a statistically significant change in fungal family (*P* < 0.05) through time or space. *n* = #/# indicates the number of sequences associated with Day1/Day21 or Day1 Absent/Day1 Present, etc. The letters (e.g., Absent_A or Day1_A) indicate a single enclosure sampled two times over the length of the experiment.

Log-normalized proportions of the CloVR-ITS heat maps of hierarchical taxonomic clusters were used to allow for a more equivalent weighting of low/high abundance taxa (White et al. 2013; Figs. 5) and statistically significant differences tested using Metastats (White et al. 2009). At the class rank, significant differences were noted at Day 1 (Eurotiomycetes), but not at Day 21 when comparing presence to absence enclosures (Fig. 3). When comparing Day 1 (eviction) to 21 (posteviction), fungal community composition significantly changed in presence (Eurotiomycetes), but not in absence enclosures (Fig. 3). A similar pattern was observed at the order and family ranks (Figs. 5). At the order rank, statistically significant changes occurred when comparing presence enclosures from Day 1-21. (Chaetothyriales, Mortierellales, Mucorales; Fig. 4). Fungal community composition did not significantly change in absence enclosures when comparing Day 1–21 (Fig. 4). Independent absence enclosures labeled A–D have overall community similarity when comparing Days 1–21 (cladograms; Fig. 4). A contrasting trend was observed in presence enclosures between Days 1 and 21 and independent presence enclosures labeled E–H (cladograms; Fig. 4). This pattern is accentuated at the family rank in presence enclosures (Fig. 5). The Lycoperdaceae and Plectosphaerellaceae, although not among the 20 most abundant families, are the exception to this pattern showing statistically significant changes in absence enclosures between Days 1 and 21 (Fig. 5). Both of the latter-mentioned families decrease in read abundance from Days 1 to 21.

### Clustering differences in fungal lineages

**Figure 6:**
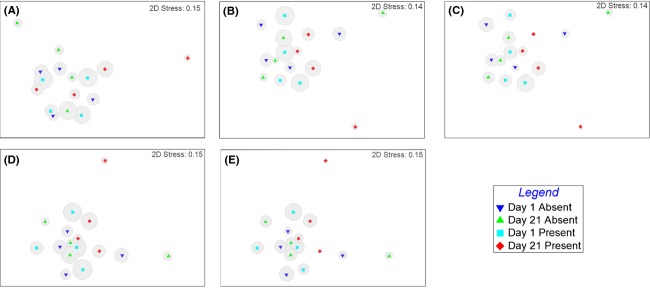
Absence/presence clustering differences using multidimensional scaling plots at Days 1 and 21. Each point represents the overall fungal community in a single enclosure. Gray bubbles represent read abundance for taxonomic groups (top 20 most abundant) indicated as statistically significant in Metastats (e.g., larger bubbles represent greater read abundance relative to smaller bubbles). (A) Fungal Class; Bubbles – Eurotiomycetes (B) Fungal Order; Bubbles – Chaetothyriales (C) Fungal Order; Bubbles – Mortierellales (D) Fungal Family; Bubbles – Herpotrichiellaceae (E) Fungal Family; Bubbles – Mortierellaceae.

The occurrence and relative abundance of fungal lineages in absence/presence enclosures at Days 1 and 21 were evaluated for their clustering differences with MDS analyses (Fig. 6). No distinct clustering patterns of overall fungal communities were observed for Days 1 and 21 presence and absence enclosures at each taxonomic rank (Fig. 6A–E). A general pattern emerges when observing read abundance of distinct taxonomic groups showing statistically significant changes in Metastats analyses; read abundance across all replicates remains consistent in absence enclosures, but significantly decreases from Days 1 to 21 across all replicates in presence enclosures (2D bubble plots in Fig. 6A–E).

## Discussion

The top-down effect has been documented in a range of different species that play part in the detritivore food web. For example, the prey consumption of forest floor spiders has been estimated (Moulder and Reichle 1972) to contribute to invertebrate abundance and leaf litter decomposition rates (Lawrence and Wise 2000, 2004; Lensing and Wise 2006). Ecological theory predicts that indirect effects occur if species (A) affects the population density of species (C) through a change in the density of a third species (B) (A > B; A > C; B > C; Billick and Case 1994; Boddy 2000). When all three species compete (e.g., complex fungal communities in soil/leaf litter), species A reduces population density of species B and C simultaneously. Competitive interactions during mycelial extension can result in the production of volatile antagonistic organic compounds or direct displacement due to interspecific interactions (Boddy 2000). These interactions will partially determine the fungal community structure and species richness (Boddy 2000). Highly competitive cord-forming basidiomycete fungi in temperate and boreal forests are thought to regulate the overall soil biodiversity (Boddy 1999; Baldrian 2008). This study was conducted in a temperate upland deciduous forest in northwest Ohio, U.S.A. Following salamander eviction, the Agaricomycetes (Basidiomycota) increased (Day 1 = 25%; Day 21 = 38.6%) and Sordariomycetes (Ascomycota) decreased (Day 1 = 19.8%; Day 21 = 10.9%) in read abundance within absence enclosures. In presence enclosures, Agaricomycete read abundance remained nearly the same (Day 1 = 14.7%; Day 21 = 12.4%), whereas the Sordariomycetes decreased (Day 1 = 12%; Day 21 = 4.1%). We hypothesize that in presence enclosures, *P. cinereus* selectively preyed upon larger invertebrates (prey-size selection; Jaeger 1990), like isopods, keeping these species at relatively consistent population numbers, thus eliminating changes in Agaricomycete abundance due to limited and selective mycoherbivory (Crowther et al. 2013). This scenario reflects a natural upland deciduous forest ecosystem but does not account for the interesting patterns observed upon salamander eviction. We hypothesized that upon salamander eviction, isopods are no longer under the predatory pressures of *P. cinereus* and are able to selectively ingest basidiomycete cords as in Crowther et al. (2013), thus reducing their abundance and opening new niches for ascomycete and zygomycete species. This hypothesis was rejected for absence enclosures, the Agaricomycetes increased, Sordariomycetes decreased, and Dothidiomycetes/Eurotiomycetes/Leotiomycetes remained nearly the same in read abundance (Table 1). Perhaps this reflects the results in Baldrian (2008) demonstrating the competitive nature of wood-inhabiting ligninolytic basidiomycetes (e.g., Agaricomycetes) in soil ecosystems and their ability to maintain and defend a territory once established.

*Plethodon cinereus* uses pheromone infused fecal pellets to establish a territorial range along the forest floor to deter and aggressively defend other salamanders from its home range (Thurow 1975; McGavin 1978; Jaeger and Gergits 1979; Jaeger 1981). The aluminum flashing enclosures used in this study mimic the territorial range and allow for complex ecosystem processes to take place that would otherwise not occur in a microcosm study. To understand the complex interactions within a detritivore food web, the microfauna, macrofauna, and predatory salamander populations (among other factors) must be taken into account. Walton et al. (2006) hypothesized that *P. cinereus* exhibit prey-size selection for larger more nutritious macrofauna like isopods (Jaeger 1990) which may release podomorphic *Collembola* (microfauna) from competition for detrital and microbial resources. Several studies have shown that fungal communities are stable and resilient enough to resist the predatory pressures of *Collembola* and other microfauna species (Parkinson et al. 1979; Whittaker 1981; Kaneko et al. 1998). Crowther et al. (2011a,b,c,d) empirically demonstrated that grazing macrofauna (isopods) exert stronger pressure on palatable basidiomycete species than other fungi leading to the assumption of top-down community control. Each of the previously mentioned studies provides a critical piece of information describing the interactions between salamanders and invertebrates, and invertebrates and fungi. Our objective here was to determine whether salamanders directly influence the composition of fungal communities to bridge the gap between upper and lower levels of the detritivore food web. In this study, Red-backed Salamanders were not found to influence overall fungal community composition or species diversity in an upland deciduous forest (two-way ANOVA; Fig. 6). The MDS plots support similar overall fungal community composition between Days 1 and 21 presence and absence enclosures. No distinct groupings were observed suggesting that *P. cinereus* does not affect the overall structure of fungal communities (Fig. 6). However, specific taxonomic groups in the Ascomycota and Zygomycota show statistically significant shifts in Metastats analyses in presence but not absence enclosures (Figs. 6). Each of these groups significantly decreases across each replicate (each enclosure) in read abundance. For example, the ascomycete groups Eurotiomycetes, Chaetothyriales, and Herpotrichiellaceae decrease in read abundance in each presence enclosure from Days 1 to 21 (Fig. 6A, B and D). The zygomycete order Mortierellales and family Mortierellaceae show the same pattern of read abundance reduction over time in the presence of a salamander (Fig. 6C and E). Removing *P. cinereus* from a natural functioning ecosystem may have cascading effects on lower trophic levels such as the micro- and macrofauna, which skews organic matter availability and allows for some fungal species to outcompete others. We hypothesize that the resident fungal communities present at Day 1, particularly the latter-mentioned ascomycete and zygomycete groups, gain a competitive edge in the absence of a keystone predator and quickly colonize the available niche space, which is represented as a lack of read reduction in absence enclosures (Figs. 6). Inversely, we hypothesize that the observed changes (read reduction) in these taxonomic groups are a result of natural competition in the presence of the keystone predator, *P. cinereus*.

The nutrient enrichment model predicts that fungi are not regulated by a top-down effect given their extensive biomass and ability to produce biochemical defenses (Moore et al. 2003). An alternative explanation to a salamander-induced structuring of distinct fungal groups is that water/solute potential (Boddy 2000), elevated CO_2_, reduced O_2_, or temperature (Chapela et al. 1988; Schoeman et al. 1996) influenced the competitive interactions of resident fungal species post-salamander eviction, resulting in the observed community shifts. Or perhaps, the salamander plays no role with respect to the fungal communities, and the results from 454 pyrosequencing are skewed due to a number of different factors (discussed in Gomez-Alvarez et al. 2009; Tendersoo et al. 2010). We acknowledge that the low amount of replicates in this study only holds limited statistical power and without looking at invertebrate abundances, only inferences on observed effects can be made. We conclude that given past studies supporting *P. cinereus* exhibiting a top-down effect on invertebrate communities, macrofauna (e.g., isopods; Crowther et al. 2013) exerting a top-down effect on fungal communities, and results from this study, that *P. cinereus* is involved in structuring specific taxonomic groups of fungi at some level. Additional replicates taken over a longer time period would be necessary to characterize this as a salamander-induced top-down effect on fungal communities. It is notoriously difficult to conserve or manage ecosystems to maintain fungal communities. The extensive distribution and biomass of *P. cinereus* have been documented in the past (Burton and Likens 1975). If salamanders are important for structuring fungal communities in a particular way, then we can use this knowledge to understand how fungal communities (and their functioning) are arranged at broad spatial scales. This study provides information linking the keystone predator *P. cinereus,* invertebrate species, and fungal communities which all play critical roles in the detritivore food web.

## Data Accessibility

Raw sequence data including .fna and .qual files were submitted to GenBank Short Reads Archive under the study accession number SRP045746.
